# Supplementary material for: Nonlocal Orbital-Free Kinetic Energy Functional from the Jellium-with-Gap Model for Finite Systems
Source: J Chem Theory Comput. 2026 Jun 12;22(13):6510–24. doi: 10.1021/acs.jctc.6c00460 (PMC13374015; doi:10.1021/acs.jctc.6c00460)
Supplement: Supplementary file 1 [file ct6c00460_si_001.pdf]

Supporting Information: Nonlocal Orbital-Free Kinetic Energy Functional from the Jellium-with-Gap Model for Finite Systems

Abhishek Bhattacharjee,[1,*] Subrata Jana,[2,†] Szymon Śmiga,[2] and Prasanjit Samal[1]

[1]*School of Physical Sciences, National Institute of Science Education and Research, An OCC of Homi Bhabha National Institute, Bhubaneswar 752050, India*

[2]*Institute of Physics, Faculty of Physics, Astronomy and Informatics, Nicolaus Copernicus University in Toruń, ul. Grudziadzka 5, 87-100 Toruń, Poland*

(Dated: June 1, 2026)

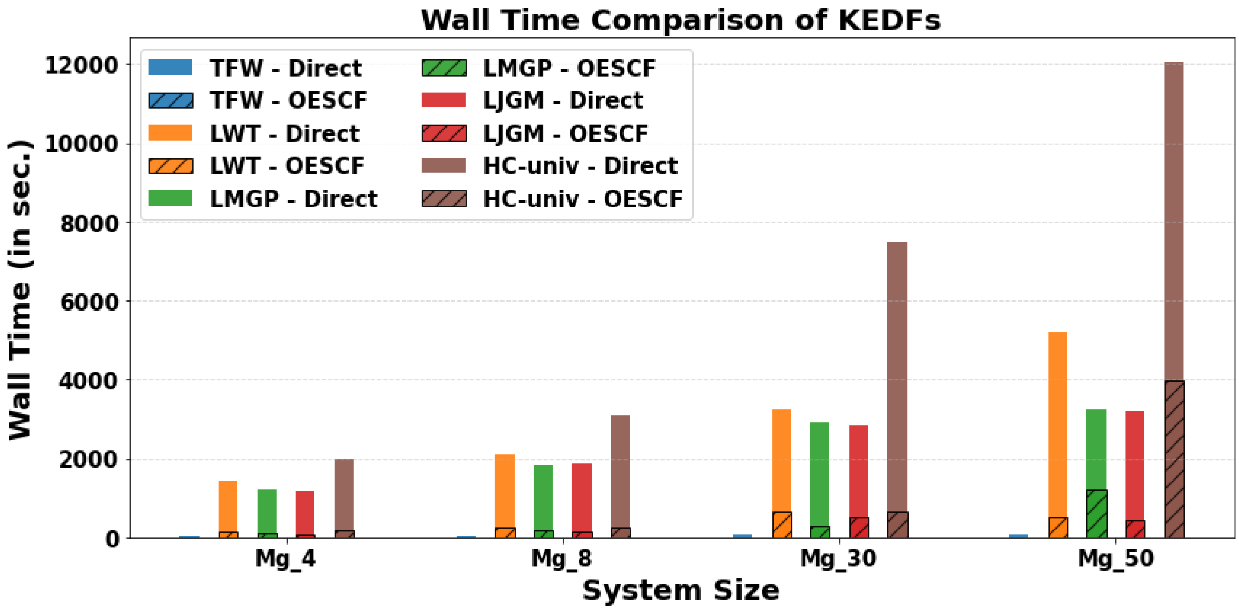

FIG. S1. Computational cost in terms of wall-time for various density-dependent NL-KEDFs corresponding to Table S5. Here we have shown two methods: (1) direct minimization and (2) the OESCF method, in both cases, with growing system size, HC becomes much more expensive or converges slowly. All calculations use a 1200 eV energy cutoff, convergence threshold 10^{-6}, and the same BLPS pseudopotential as mentioned in main text.

TABLE S1. Energies (in eV) of different clusters as obtained from different methods. Here LJGM7 refers to LJGM with E_g=0.07 eV.

Clusters	KS	TFvW	LWT	HC	LMGP	LJGM000	LJGM005	LJGM007	LJGM01
Mg_8	-23.175	-20.880	-21.990	-23.330	-23.400	-23.587	-23.230	-23.089	-22.859
Mg_{30}	-23.226	-21.710	-22.350	-23.345	-23.400	-23.535	-23.100	-22.926	-22.642
Mg_{50}	-22.289	-20.710	-21.500	-22.429	-22.580	-22.578	-22.170	-22.003	-21.737
Li_8	-5.707	-4.729	-5.179	-5.784	-5.800	-5.902	-5.711	-5.631	-5.505
Li_{30}	-6.633	-6.088	-6.317	-6.666	-6.713	-6.734	-6.574	-6.506	-6.398
Al_8	-52.867	-47.041	-53.319	-53.280	-53.319	-53.927	-52.800	-52.327	-53.927
Si_8	-105.909	-96.580	-100.370	-106.394	-107.460	-107.463	-105.470	-104.635	-103.338
Si_{30}	-107.419	-100.840	-103.720	-107.723	-108.390	-108.392	-106.910	-106.304	-105.353
Ga_4As_4	-116.137	-106.450	-110.560	-116.905	-117.690	-118.045	-116.200	-115.428	-114.219
In_4P_4	-112.592	-101.460	-106.040	-112.971	-114.170	-114.173	-112.360	-111.612	-110.445
Al_4Sb_4	-93.265	-84.330	-88.170	-93.945	-94.910	-94.915	-93.010	-92.221	-91.402
Al_4P_4	-115.981	-105.880	-116.139	-116.241	-116.139	-117.310	-115.191	-114.302	-117.310
Ga_4P_4	-118.614	-108.088	-119.296	-119.183	-119.296	-120.419	-118.582	-117.813	-120.419
Ga_4Sb_4	-95.141	-85.660	-95.701	-95.645	-95.701	-96.657	-94.835	-94.073	-96.657
In_4As_4	-109.041	-98.321	-109.551	-109.387	-109.551	-110.585	-109.079	-108.452	-110.585
In_4Sb_4	-93.482	-83.986	-93.637	-93.531	-93.637	-94.468	-92.815	-92.124	-94.468

* abhishek.bhattacharjee@niser.ac.in
† subrata.jana@umk.pl, subrata.niser@gmail.com

TABLE S2. Density error D_0 of different clusters as obtained from different methods. Here LJGM7 refers to LJGM with E_g=0.07 eV.

Clusters	TFvW	LWT	HC	LMGP	LJGM000	LJGM005	LJGM007	LJGM01
Mg_8	20.951	9.170	5.955	5.947	5.947	5.772	5.850	6.154
Mg_{30}	13.834	6.851	3.876	3.974	3.974	3.769	3.760	3.855
Mg_{50}	12.490	6.601	3.533	3.841	3.841	3.638	3.589	3.573
Li_8	15.614	9.740	9.185	8.695	8.695	9.200	9.154	9.403
Li_{30}	12.487	6.321	4.513	4.407	4.353	4.266	4.333	4.655
Al_8	15.822	8.845	6.030	6.395	6.395	6.355	6.229	7.359
Si_8	17.787	9.634	6.187	6.262	6.262	6.201	6.254	6.394
Si_{30}	15.108	8.037	3.532	3.737	3.737	3.552	3.508	3.464
Ga_4As_4	20.189	9.556	7.005	6.940	6.940	6.941	6.979	7.142
In_4P_4	22.382	12.781	6.988	6.827	6.827	6.818	6.888	7.053
Al_4Sb_4	22.030	12.748	7.549	7.342	7.342	7.257	7.273	7.374
Al_4P_4	19.805	10.073	5.154	5.022	5.022	4.959	5.033	4.969
Ga_4P_4	21.689	11.647	6.747	6.708	6.708	6.693	6.733	6.748
Ga_4Sb_4	19.901	9.919	5.947	5.837	5.837	5.762	5.783	5.884
In_4As_4	20.002	10.169	6.471	6.422	6.422	6.288	6.380	6.202
In_4Sb_4	20.755	10.827	5.903	5.862	5.862	5.687	5.775	5.664

TABLE S3. Median Absolute Relative Percentage Errors (MARPEs) and Mean Absolute Relative Percentage Errors (MeanARPEs) of various functionals for semiconductors and metals test set.

		TFvW	LWT	LMGP	LJGM0	LJGM	LJGM1	HC
**MARPE**	**Energy**	3.76	3.70	0.90	1.46	**0.32**	1.90	0.49
	**Density**	10.93	9.69	5.94	5.95	**5.77**	6.15	5.95
**MeanARPE**	**Energy**	3.96	3.55	1.52	2.06	**0.78**	2.16	1.08
	**Density**	10.26	9.55	6.71	6.78	**6.63**	6.74	6.77

TABLE S4. Median Absolute Relative Percentage Errors (RMAREs) and Mean Absolute Relative Percentage Errors (RMeanAPEs) of various functionals.

**Functional**	TFvW	LWT	LMGP ($A = 0.2$)	HC ($\lambda = 0.01177$, $\beta = 0.7143$)	LJGM0 ($E_g = 0$)	LJGM ($E_g = 0.05$)	LJGM1 ($E_g = 1.0$)
RMARE	7.13	6.67	2.28	1.71	3.09	**1.42**	3.75
RMeanARE	7.48	5.92	2.32	1.77	3.07	**1.50**	3.71

TABLE S5. Total computational wall time (in seconds) for evaluating the kinetic potential using various KEDFs with the OESCF method. The number of SCF cycles to convergence is shown **in parentheses**. All calculations use a 1200 eV energy cutoff, convergence threshold 10^{-6}, and the "Mg_OEPP_PZ.UPF" pseudopotential.

	**Direct Minimization**				**OESCF Method**			
**Functional**	Mg_4	Mg_8	Mg_{30}	Mg_{50}	Mg_4	Mg_8	Mg_{30}	Mg_{50}
TFW	20(90)	33(90)	63(91)	65(82)	—	—	—	—
LWT	1418(88)	2106(91)	3247(92)	5197(140)	134(9)	251(12)	646(17)	505(13)
LMGP	1204(86)	1845(87)	2910(85)	3244(85)	110(6)	164(7)	483(9)	1224(19)
LJGM	1177(86)	1865(87)	2831(85)	3211(85)	73(6)	135(7)	518(11)	441(8)
HC	1385(103)	2179(105)	4432(131)	8186(201)	184(6)	313(7)	483(10)	621(9)
HC-univ	1981(131)	3077(141)	7495(238)	12058(313)	170(7)	236(7)	638(8)	3987(38)
